# Silymarin: A Novel Natural Agent to Restore Defective Pancreatic β Cells in Streptozotocin (STZ)-induced Diabetic Rats

**Published:** 2016

**Authors:** Amir Amniattalab, Hassan Malekinejad, Aysa Rezabakhsh, Shirin Rokhsartalab-Azar, Shahin Alizade-Fanalou

**Affiliations:** a *Department of Pathology, Islamic Azad University, Urmia Branch, Urmia, Iran. *; b *Department of pharmacology and toxicology, Faculty of Veterinary medicine, Urmia University, Iran. *; c *Department of pharmacology and toxicology, Faculty of Pharmacy, Urmia University of Medical Sciences, Urmia, Iarn. *; d *Department of Pharmacology and toxicology, Faculty of Pharmacy, University of Medical sciences, Tabriz, Iran.*

**Keywords:** Antioxidant status, Cell restoring, Melatonin, Silymarin, Streptozotocin-induced diabetes

## Abstract

This study aimed to investigate the potency of silymarin (SMN) and melatonin (MEL) on restoring the pancreatic   cells in streptozotocin (STZ)-induced diabetic rats. Male Wistar rats were divided into five groups, including: control (C), untreated diabetic (D), SMN-treated diabetic (50 mg/Kg, orally), MEL-treated diabetic (10 mg/Kg, i.p.), and SMN plus MEL-treated diabetic rats. Diabetes was induced by injection of STZ (50 mg/Kg, i.p.). The blood glucose and insulin levels were measured. After the 28 days treatment period, antioxidant status was analyzed by determination of total antioxidant capacity (TAC) in the liver and serum. The histopathological changes in the pancreatic islets were examined by histochemical staining and enumeration of   cells. Although none of the test compounds reduced the blood glucose level to normal concentration, however SMN alone and in combination with MEL was able to decline it significantly (P<0.05) after 28 days administration. Both SMN and MEL could recover the diabetes-reduced TAC values. Moreover, the diabetes-induced cellular vacuolation and   cells depletion were improved by the SMN treatment. Our data suggest that the SMN and MEL treatment was able to normalize the antioxidant status, while only SMN administration could restore the  cells of Langerhans islets in diabetic rats.

## Introduction

Type 1 diabetes mellitus (T_1_DM) is known as a worldwide endocrine disorder characterized by insulin insufficiency and hyperglycemia due to destruction of the pancreatic beta cells (1). Once the damage to β-cells exceeded from threshold, the pancreas is not able to secrete a sufficient amount of insulin and consequently due to high blood glucose diabetes mellitus would be inevitable. In addition, overproduction of reactive oxygen species (ROS) has been reported in T_1_DM. Free radicals or oxygen-derived species which originate from physiologic cellular reactions, inflammatory reactions and cell injury play an important role in the pathogenesis and complications of diabetes mellitus. In diabetes increased ROS levels lead to repression of insulin synthesis through enhancing apoptosis of pancreatic beta-cells. There are increasing data indicating an enhanced oxidative stress and changes in antioxidant capacity in both clinical and experimental forms of diabetes mellitus, suggesting one of the key mechanisms in the pathogenesis of secondary diabetic complications (2). On the other hand, hyperglycemia can also induce oxidative stress by various mechanisms and reduces antioxidant capacity.

During the last decade much attention has been paid on using plant-derived materials including various forms of extracts as an antioxidant source to eliminate excessive ROS (3-6). Silymarin (SMN), the active components of milk thistle extract, is a polyphenolic flavonoid complex that has long been extensively used in patients suffering from liver diseases. Other biological activities of SMN such as anti-inflammatory, antioxidant, and anti-cancer have been demonstrated both in *in-vitro* and *in-vivo* studies (7-9).

To induce diabetes experimentally in the rodents, using chemicals which selectively destroy pancreatic beta cells, is the easiest and convenient approach. One of the most used substances to induce diabetes in the rat is streptozotocin (STZ), which dose-dependently produce diabetes. In STZ-induced model of type-I diabetes, the increased oxidative stress along with pancreatic-cell damage have been well documented (9). 

In our previous study we showed that SMN can recover the hepatic glycogen resources in hepatocytes of diabetic rats (10). Other pre-clinical and clinical reports also support the anti-diabetic potential of silymarin (11). However, there is lack of knowledge about the direct effect of SMN on the pancreatic beta cells in diabetic cases. The current study was aimed to investigate the effect of SMN on damaged pancreatic beta cells in the STZ- induced diabetic rats. Moreover, to compare the anti- diabetic effect of SMN, melatonin as endogenously released antioxidant was also included in the present study. 

## Experimental

*Materials and methods*

*Chemicals*

Silymarin (SMN, S 0292), Streptozotocin (STZ, S0130) and melatonin (MEL, M5250) standards were purchased from Sigma-Aldrich (Germany). 2, 4, 6-tri-2-pyridyl-1, 3, 5-triazin (TPTZ), FeCl_3_, dimethyl sulfoxide (DMSO) and ethanol were obtained from Merck (Germany). All other chemicals were commercial products of analytical grade. 

*Experimental Design*

Thirty male and clinically healthy Wistar-Albino rats (220-250 g) were kept under a 12-h light cycle with free access to a standard diet and water in a temperature controlled room at Urmia University. The animals randomly assigned to five groups (n = 6) including control, non-treated diabetic and treated diabetic. The treated diabetic group subdivided into three groups which received SMN (50 mg/Kg, p.o.), MEL (10 mg/Kg, i.p.) and/or a combination of SMN and MEL for 28 consecutive days. Control rats were only exposed to the same amount of citrate buffer as the solvent of STZ.

*Induction of Type 1 Diabetes*

Type 1 DM was induced by a single (i.p.) injection of a freshly prepared solution of STZ in 0.01 M citrate buffer, pH 4.5, (50 mg/Kg/BW). Rats with a blood glucose level of 250 mg/dL or greater were accepted as diabetic and included in this study. For monitoring the blood glucose (BG), blood samples were drawn from the tail veins of rats on days of 1, 2, 3, 7, 14, 21, 28 and the glucometer set was used (Elegance, CT-X10, Convergent Technologies GmbH & Co.KG, Marburg, Germany). 

*Preparation of Serum Samples and Tissue collection*

At the end of experiment all rats were anesthetized by using diethyl ether. Blood samples were taken from the heart of animals. Serum samples were obtained via centrifuging of blood samples at 3,000 × g for 10 min at 4 °C. Afterward, the rats were euthanized by inhalation of CO_2_. The liver and pancreases were removed and after trimming the adipose tissues from pancreases and washing the liver samples with chilled saline normal, the pancreases were fixed in 10% formalin for further histopathological examinations and the liver samples following snap freeze in liquid nitrogen were kept at -70 °C for further biochemical analyses. 

*Assessment of total antioxidant capacity (TAC)*

To determine the effect of diabetes on total antioxidant capacity and consequently potential therapeutic effect of SMN and MEL on disturbed antioxidant system, the reducing capacity of serum and liver homogenate were measured. The assessment carried out based on ferric reduction antioxidant power (FRAP) assay (12). Briefly, at low pH which was provided using acetate buffer (300 mM, pH 3.6), reduction of Fe^III^-TPTZ (2, 4, 6-tri-2-pyridyl-1, 3, 5-triazin, Merck, Germany) complex to the ferrous form produces an intensive blue color that could be measured at 593 nm. The intensity of the complex following adding the appropriate volume of the serum/or the liver tissue homogenate supernatant to reducible solution of Fe^III^-TPTZ is directly related to total reducing power of the electron donating antioxidant. Aqueous solution of Fe^II ^(FeSO4.7H2O) and appropriate concentration of freshly prepared ascorbic acid were used as blank and standard solutions, respectively. 

*Insulin measurement*

Serum level of insulin was measured by 1-step chemiluminescence sandwich assay using directly coated magnetic microparticles (LIAISON® Insulin, DiaSorin S.p.A. via Crescentino 13040 Saluggia, Vercelli, Italy) and according to instructions of kit manufacturer. The lowest level of detection was 0.23-0.61 µIU/mL and measuring range was 0-500 µIU/mL. 

*Histopathological and histochemicaal examinations*

The Hematoxyline & Eosin staining was performed on the pancreas sections (5 µM) to evaluate any diabetes-induced histological damages. For each animal in the test and control groups at least three slides were prepared. 

To clarify the diabetes and test compounds effect on beta cells, the aldehyde fuchsin histochemical method was also performed (13). By this staining beta cells in islets of pancreas are stained purple-violet. In order to enumerate the number of beta cells, for each study group, 10 equally same size microscopic fields were screened and the cells with purple-violet granules counted as beta cells. The pathological examinations were performed by a pathologist who was completely unaware of the study purposes. 

*Statistical analysis*

The obtained numerical data were analyzed using Graph Pad Prism software (version 2.01. Graph Pad software Inc. San Diego, California). The comparisons between groups were made by analysis of variance (ANOVA) followed by Bonferroni *post hoc* test. A P value <0.05 was considered significant.

## Results

Blood glucose level in all experimental groups was measured before, during the experiment and after the treatment with various test compounds. The collected data indicate that the experimentally-induced diabetes resulted in a remarkable elevation of blood glucose level in the animals. In contrary to SMN, which after 28 days administration was able to lower significantly (P<0.05) the blood glucose level –compared to glucose level before the treatment-, MEL alone was not able to alter the increased level of blood glucose significantly (P>0.05). It is interesting to be noted that MEL in combination with SMN could lower slightly the blood glucose level (Table 1.). Moreover, only SMN lowered the blood glucose level significantly (P<0.05) in comparison to the non-treated group and when it was compared against the blood glucose level before the treatment.

At the end of treatment, insulin level of blood was also measured and the results showed that the experimentally-induced diabetes lowered significantly (P<0.05) the insulin level. Although all three treated diabetic groups showed an increase in insulin level but only those animals, which received SMN alone and/or SMN and MEL, showed significant (P<0.05) increase of insulin level. The highest level of blood insulin level between the treated diabetic groups was found in the group which was treated with SMN alone (Table 1.). 

**Figure 1:**
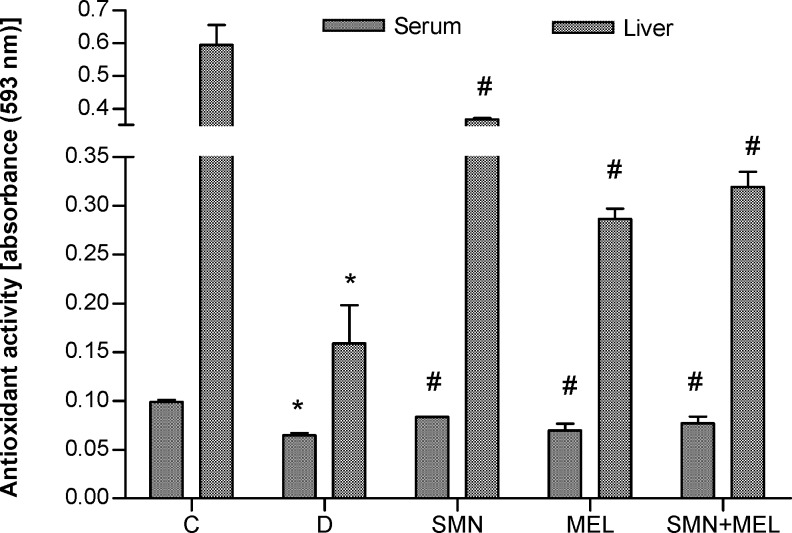
Effect of SMN and MEL on total antioxidant capacity in diabetic animals

To demonstrate the antioxidant effect of both given compounds the total antioxidant capacity was measured in serum and the liver tissue homogenate. Results showed that diabetes remarkably reduced the TAC both in the liver and serum. On the other hand, both test substances in individual and in combination forms were able to recover the diabetes-reduced antioxidant capacity in the liver. MEL alone failed to recover the diabetes-reduced TAC in serum (Figure 1.). 

**Figure 2:**
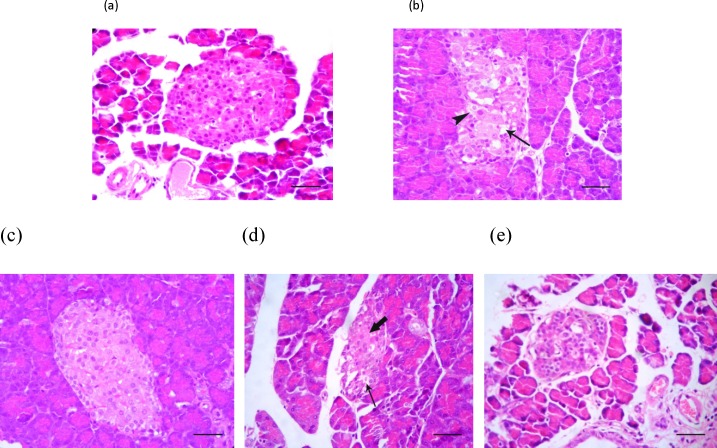
Photomicrograph of the rat's pancreas: (a) Normal appearance of cells inside the Langerhans islets, (b) cellular vacuolation (arrow) and loss of cytoplasmic tonality (arrowhead) in some cells of Langerhans islets in the untreated diabetic rats, (c) Normal appearance of endocrine pancreatic cells in Langerhans islets in the SMN-received rats, (d) MEL-received diabetic rats showing cellular depletion (thick arrow) and vacuolation (thin arrow) in cells of Langerhans islets, (e) pancreas tissue from diabetic animals, which received both test compounds, representing cellular aggregation in Langerhans islets which is more than MEL-received group and lesser than the control group. H&E (100 X) and scale bars = 25 µM

To examine the impact of diabetes on the pancreas histology, firstly the H&E staining was conducted and the pathological findings indicated the cellular vacuolation and loss of cytoplasmic tonality in the diabetic non-treated animals (Figure 2-b.). Interestingly those diabetic animals that received SMN alone, demonstrated approximately closer histological feature of Langerhans islets to the control group (Figure 2-a, 2-c). The cellular depletion and vacuolation in MEL-treated and cellular aggregation in Langerhans islets of SMN + MEL-received animals were found (Figure 2-d, 2-e). 

**Figure 3:**
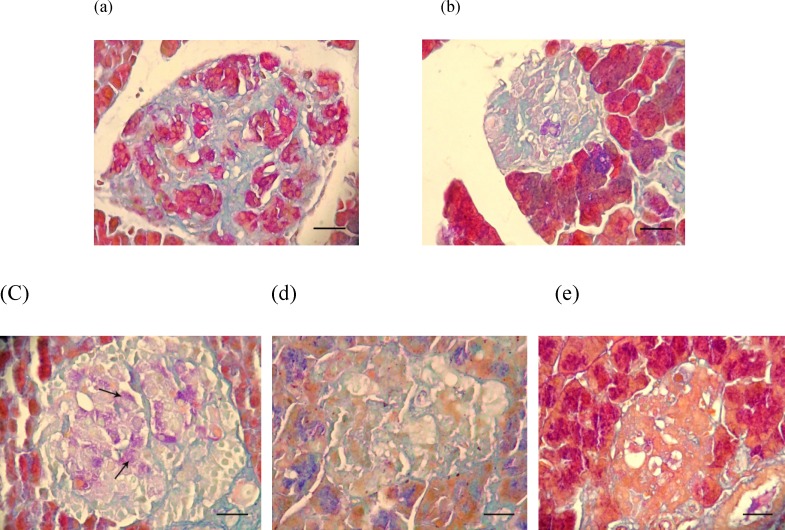
Photomicrograph of the rat's pancreas: (a) normal beta cells in Langerhans islet containing purple granules, (b) destruction and vacuolation (arrowhead) of beta cells and deficiency of their cytoplasmic tonality in the untreated diabetic rats, (c) there are few purple granules containing cells (arrows) in Langerhans islets in the SMN-received rats , (d) MEL-received diabetic rats are showing cellular depletion (thick arrow) and vacuolation (thin arrow) in cells of Langerhans islets similar to the non-treated diabetic rats, (e) pancreas tissue from diabetic animals, which received both test compounds with very few purple granules containing cells in Langerhans islets. Aldehyde fuchsin histochemical staining (100 X) and scale bars = 25 µM

To confirm the found histopathological lesions, specific staining of pancreatic beta cells was performed and the results (Figure 3.) showed that indeed SMN alone was able to restore the beta cells which characterized with purple violet granules (Figure 3-c). In histochemical staining of the pancreas tissue from those diabetic animals, which were co-administered SMN and MEL, a weak positive staining of beta cells was demonstrated (Figure 3-e). 

**Figure 4:**
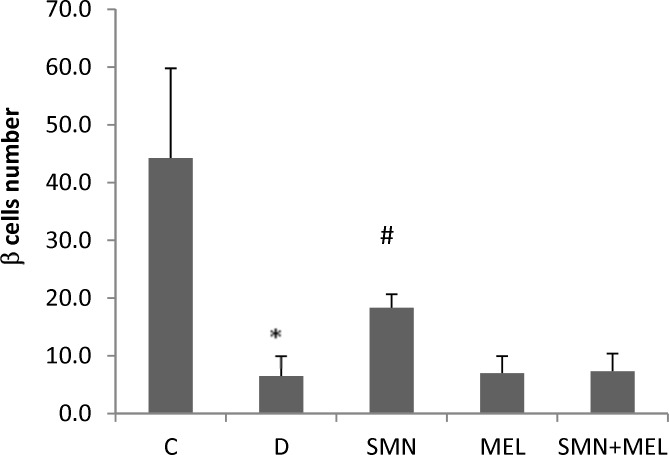
Effect of SMN and MEL on the number of beta cells in Langerhans islet of diabetic animals

The beta cells number was enumerated in 10 randomly selected microscopic fields in all study groups and as illustrated in Figure 4, the STZ-induced diabetes resulted in a significant (P<0.05) reduction of beta cells. Among the treated diabetic groups, only the SMN-received animals showed a significant restoring of beta cells (Figure 4.). 

**Table 1:** Changes in blood glucose and insulin levels of animals (Mean ± SD; n=6)

**Groups**		**Blood glucose (mg/dL)**	
**Insulin** ** (µ** **IU/mL)**		**Start**	**End**
C	7.7 ± 0.2	134 ± 13.3a	113 ± 21.7^a^
D	3.1 ± 0.4^*^	482.3 ± 58.6*^a^	473.0 ± 31.1^*^^a^
S	5.6 ± 0.4 #	530.6 ± 65.5^a^	406.3 ± 29.4#^b^
M	3.4 ± 0.5	471.0 ± 56.9^a^	518.7 ± 30.7^a^
S+M	4.1 ± 0.2 #	528.3 ± 44.6^a^	456.0 ± 12.3^b^

C: control, D: Diabetic group, S: Silymarin-treated diabetic animals, M: Melatonin-treated diabetic rats and S+M: the diabetic animals, which received a combination of silymarin and melatonin. Stars are showing significant differences between the control and untreated diabetic groups and

# s are representing significant differences (P<0.05) between untreated and treated diabetic groups. Different superscript letters

(a and b) are indicating significant differences between the blood glucose levels before and after the treatment period at the same group.

## Discussion

The current study showed that SMN but not MEL was able to restore the STZ-damaged pancreatic β cells, which consequently resulted in a remarkable elevation and reduction of blood level of insulin and glucose, respectively. Additionally, antioxidant status evaluation in the liver and serum of animals revealed that both compounds in individual and combination forms were able to recover the diabetes-reduced total antioxidant capacity. 

There are increasing data indicating a crucial role of oxidative stress in the pathogenesis of the diabetes-induced complications. Oxidative stress not only is one of the causative factors in the induction of diabetes but also is induced in the diabetic patients by various mechanisms including increased advanced glycation end-products, increased polyol pathway flux, glucose autoxidation and mitochondrial overproduction of superoxide (14, 15). Besides, in diabetes extra free fatty acids via generation of acetyl-Co A and NADH leads to extra mitochondrial superoxide production.

It has been well established that STZ by methylation of DNA of the pancreatic beta cells results in DNA fragmentation. On the other hand, it has been shown that any compound which can inhibit the DNA methylation such as nicotinamide will be able to protect beta cells from the toxic effect of STZ and to prevent the development of diabetes (16). Alternative mechanisms for the STZ-induced diabetes related to its ability to act as a strong donor of NO and also its ROS generation capacity (17). To test the role of oxidative stress in the pathogenesis of STZ-induced diabetes complications, the total antioxidant capacity was determined in serum and the liver. Our results indicate that although both compounds could recover the diabetes-reduced antioxidant capacity in the liver and serum however SMN could elevate the TAC values higher than that MEL**. **The antioxidant effects of both compounds have been well documented. Melatonin as an endogenous substance crosses all biological membranes and is able to exert its antioxidant effects such as free radical scavenging activity receptor-independently. It has been however shown that MEL stimulates some antioxidant enzyme indirectly and via receptor binding pathway, too (18, 19).

On the other hand, SMN as a well known antioxidant substance acts as regulator of the intracellular glutathione, cell membrane stabilizer, free radical scavenger, antioxidant enzymes stimulator and anti-lipid peroxidation (10, 20). Despite the fact that both compounds could significantly recover the antioxidant capacity in serum and the liver, however the diabetes-induced complications including blood glucose and insulin levels were remained high and low respectively in the MEL-treated diabetic rats. By contrast both glucose and insulin levels remarkably were reduced and elevated in the groups of diabetic animals, which received SMN either alone or in combination with melatonin, indicating that SMN may exerts its antidiabetic effect via other pathways than having only antioxidant effect. 

To discover how SMN but not MEL could significantly increase the insulin level and consequently reduce the blood glucose level, we examined the pancreas tissue using simple staining method of H&E. our findings demonstrated that SMN could improve the diabetes-induced cellular vacuolation and loss of cytoplasmic tonality, while MEL-received diabetic rats illustrated cellular depletion and vacuolation in cells of Langerhans islets. These findings were not fair enough to explain all the diabetes-induced complications such as insulin and glucose level changes, nevertheless, the SMN protective effect on diabetes-induced disorders at least partly could be concluded. In order to uncover the insulin producing cells fate in the groups of diabetic animals that were treated with various compounds, the special staining and enumeration for beta cells were conducted and interestingly only the SMN-treated diabetic rats showed beta cells presence in the islets, suggesting either restoring the beta cells or protecting them from the STZ-induced damages. 

There could be several approaches to be considered: whether SMN was able to stimulate the potentially present stem cells to be converted into functionally active beta cells and/or SMN protected some portion of beta cells from STZ-induced damages. Previous studies showed the possibility of beta cells neogenecity in adult mouse islets that consequently resulted in insulin formation and glucose level regulation (21). Another possibility could be the SMN capability to stimulate the beta precursor cells to differentiate into insulin forming cells (22). Similar to our finding, previous reports showed that insulin administration to the STZ-treated rats stimulated the recruitment of new beta cells from a precursor population (23). 

Our data also showed that co-administration of SMN along with MEL, not only was not resulted in a synergistic effect in terms of beta cells restoring and insulin secretion, but the SMN exerted effect in individual form also diminished in the combination therapy. There could be two possible explanations for this finding; including probable incompatibility between two compounds and also possible pro-oxidant effect of the combination therapy. It is thought that in the combination therapy, an excess level of antioxidant compounds revers their safe and therapeutic effects to toxic and pro-oxidant action. There are increasing data indicating that being antioxidant or pro-oxidant mainly related to the concentration of used substances. The concentration-dependent antioxidant/pro-oxidant activity of curcumin, vitamin E and vitamin C has been documented (24, 25). 

## Conclusion

These data indicate that SMN individually could slightly reduce the glucose level and increase the insulin level, which supported by the increased number of beta cells presence in the STZ-induced diabetic rats. However, MEL neither alone nor in combination with SMN could improve the diabetes related complications. These findings are primary data that absolutely need to be confirmed with further mechanistic studies to highlight the possible implication for protective usage of SMN along with other glucose lowering chemicals. 
